# Decreased Nuclear Ascorbate Accumulation Accompanied with Altered Genomic Methylation Pattern in Fibroblasts from Arterial Tortuosity Syndrome Patients

**DOI:** 10.1155/2019/8156592

**Published:** 2019-01-13

**Authors:** Csilla E. Németh, Zsófia Nemoda, Péter Lőw, Pál Szabó, Erzsébet Z. Horváth, Andy Willaert, Annekatrien Boel, Bert L. Callewaert, Paul J. Coucke, Marina Colombi, Gábor Bánhegyi, Éva Margittai

**Affiliations:** ^1^Department of Medical Chemistry, Molecular Biology, and Pathobiochemistry, Semmelweis University, Budapest 1094, Hungary; ^2^Department of Anatomy, Cell and Developmental Biology, Eötvös Loránd University, Budapest 1117, Hungary; ^3^Research Centre for Natural Sciences of the Hungarian Academy of Sciences, Budapest 1117, Hungary; ^4^Center for Medical Genetics, Ghent University, Ghent B-9000, Belgium; ^5^Division of Biology and Genetics, Department of Molecular and Translational Medicine, University of Brescia, Brescia 25123, Italy; ^6^Pathobiochemistry Research Group of Hungarian Academy of Sciences & Semmelweis University, Budapest 1094, Hungary; ^7^Institute of Clinical Experimental Research, Semmelweis University, Budapest 1094, Hungary

## Abstract

Ascorbate requiring Fe^2+^/2-oxoglutarate-dependent dioxygenases located in the nucleoplasm have been shown to participate in epigenetic regulation of gene expression via histone and DNA demethylation. Transport of dehydroascorbic acid is impaired in the endomembranes of fibroblasts from arterial tortuosity syndrome (ATS) patients, due to the mutation in the gene coding for glucose transporter GLUT10. We hypothesized that altered nuclear ascorbate concentration might be present in ATS fibroblasts, affecting dioxygenase activity and DNA demethylation. Therefore, our aim was to characterize the subcellular distribution of vitamin C, the global and site-specific changes in 5-methylcytosine and 5-hydroxymethylcytosine levels, and the effect of ascorbate supplementation in control and ATS fibroblast cultures. Diminished nuclear accumulation of ascorbate was found in ATS fibroblasts upon ascorbate or dehydroascorbic acid addition. Analyzing DNA samples of cultured fibroblasts from controls and ATS patients, a lower global 5-hydroxymethylcytosine level was found in ATS fibroblasts, which could not be significantly modified by ascorbate addition. Investigation of the (hydroxy)methylation status of specific regions in six candidate genes related to ascorbate metabolism and function showed that ascorbate addition could stimulate hydroxymethylation and active DNA demethylation at the PPAR-*γ* gene region in control fibroblasts only. The altered DNA hydroxymethylation patterns in patient cells both at the global level and at specific gene regions accompanied with decreased nuclear accumulation of ascorbate suggests the epigenetic role of vitamin C in the pathomechanism of ATS. The present findings represent the first example for the role of vitamin C transport in epigenetic regulation suggesting that ATS is a compartmentalization disease.

## 1. Introduction

Beyond its antioxidant properties, ascorbic acid (AA) [1]—the reduced form of vitamin C—is required for the proper functioning of several enzymes, including Fe^2+^/2-oxoglutarate-dependent dioxygenases [2] present in various subcellular compartments. Fe^2+^/2-oxoglutarate-dependent dioxygenases catalyze diverse oxidative reactions including the posttranslational modification of proteins in the endoplasmic reticulum (ER) and demethylations related to epigenetic regulation in the nucleus. Nucleoplasmic JmjC-domain-containing demethylases [3] and TET (ten-eleven translocation) methylcytosine dioxygenases [4] promote the demethylation of histones and DNA, respectively, both mechanisms being involved in the epigenetic regulation of transcription [5]. 5-Methylcytosine (5-mC) demethylation occurs via consecutive oxidative steps catalyzed by the TET dioxygenases through a 5-hydroxymethylcytosine (5-hmC) intermediate [6].

To ensure the AA supply of these AA-dependent enzymes in various subcellular compartments, transporters are needed in the membrane of organelles. In general terms, AA transport is mediated by sodium-dependent transporters, while its oxidized form dehydroascorbic acid (DHA) is transported by GLUT family transporters [7, 8]. However, the occurrence and functioning of these transporters have been hardly elucidated in the endomembranes [9].

Arterial tortuosity syndrome (ATS, OMIM #208050) is a monogenic autosomal recessive connective tissue disorder characterized by elongation and generalized tortuosity of the major arteries. ATS is caused by the loss-of-function mutations in *SLC2A10* gene encoding GLUT10 protein, a member of the glucose transporter family GLUT [10–14]. However, the exact role of GLUT10 in the ATS pathogenesis remains still debated. The perinuclear location of GLUT10 was reported in fibroblasts [12], which has been confirmed by forthcoming studies [15, 16]. These observations raised the possibility that the compromised transport of glucose into the nucleoplasm can be responsible for the pathogenesis [12]. However, recently we demonstrated that GLUT10 functions as a DHA transporter, carrying the oxidized form of vitamin C through the perinuclear membrane. This was proven by measuring the transport activity of *in vitro*-translated GLUT10 protein incorporated in liposomes and by detecting a diminished DHA uptake through the endomembrane system of fibroblasts derived from ATS patients [17].

Based on the abovementioned findings, we hypothesized that the activity of nucleoplasmic Fe^2+^/2-oxoglutarate-dependent dioxygenases is lower in the ATS patients' fibroblasts due to the diminished transport of DHA and the consequent impoverishment of the nucleoplasm in the vitamin. Thus, experiments were undertaken to investigate the subcellular distribution of AA and DHA and the possible DNA (hydroxy)methylation alterations in fibroblasts derived from ATS patients. According to our assumption, decreased nuclear accumulation of AA and altered DNA hydroxymethylation patterns at both the global level and at specific gene regions were found in patients' fibroblasts, which suggests the epigenetic role of vitamin C in the pathomechanism of ATS.

## 2. Materials and Methods

### 2.1. Cell Cultures

Skin fibroblasts from four ATS patients and four healthy donors were established from skin biopsies as previously reported [12]. Patients were either homozygous for the c.1411+1G>A splicing mutation [11] or were bearing the homozygous c.510G>A mutation [12]. Three additional skin fibroblast primary culture samples from control subjects were obtained at the Institute of Genomic Medicine and Rare Disorders, Semmelweis University (Budapest, Hungary). One postmortem dermis tissue was also used (from the 2nd Department of Pathology, Semmelweis University). The study was approved by the medical ethical committee of the University Hospital Spedali Civili of Brescia, Italy, the Ghent University Hospital, Belgium, and the Semmelweis University and was performed in accordance with the Declaration of Helsinki Principles.

Fibroblast cells were cultured in Dulbecco's Modified Eagle Medium supplemented with 10% fetal bovine serum, 1% penicillin-streptomycin, and 1% minimum essential medium nonessential amino acids (Life Technologies, Carlsbad, CA, USA). Cell cultures were grown *in vitro* at 37°C and in a 95% air and 5% CO_2_ atmosphere. Cells were preincubated with 50 *μ*M of AA (final concentration) for 24 hours before harvesting them. Cells were analysed at the same *in vitro* passage number (5th to 7th).

### 2.2. Cell Treatment

Fibroblasts were treated with AA or DHA as indicated. DHA is an unstable compound at physiologic pH and temperature, with a half-life of minutes; via hydrolysis of the lactone ring, it is irreversibly converted to 2,3-diketo-1-gulonic acid [18]. Thus, the addition of DHA cannot ensure a stable, prolonged extracellular concentration of the compound. Therefore, with the exception of certain short-term experiments, AA was added to the cells. According to present knowledge, the transportable form of the molecule within the cell is DHA, as endomembranes do not possess AA transporters [9, 7] and oxidation of AA greatly enhances the transport across the ER membrane [19]. Since AA-dependent reactions—essentially present in all cellular compartments—lead to the direct or consequential formation of DHA within the cell, DHA transport through the endomembranes becomes possible. Due to these reasons, subcellular distribution of AA and the efficiency of AA-dependent reactions mainly reflect the extent of intracellular DHA transport. Because of the latter reason and of the instability of DHA, AA was used in our long-term experiments.

### 2.3. Transmission Electron Microscopy and Immunoelectron Microscopy

Transmission electron microscopy was performed as previously described [20]. After pretreatment of the cells with either 50 *μ*M (final concentration) of AA or DHA for 5 min, the media were removed from confluent cell cultures, cells were washed with phosphate-buffered saline (PBS), and trypsin was then added to make the cells detach from the bottom of the flask. After five minutes of incubation at 37°C, the reaction was stopped with a trypsin inhibitor. Then, cells were resuspended in PBS, washed briefly, and collected by centrifuge. Pellets were fixed in 3.2% paraformaldehyde, 0.5% glutaraldehyde, 1% sucrose, and 0.028% CaCl_2_ in 0.1 M sodium cacodylate, pH 7.4, overnight at 4°C, and embedded into London Resin Gold (Agar Scientific, UK) without postfixation according to the manufacturer's recommendations.

Ultrathin sections (90 nm) were collected on nickel grids and incubated with rat polyclonal antibody to conjugated Vitamin C (Abcam) (1 : 25) in PBS with 3% milk overnight at 4°C followed by 18 nm Colloidal Gold-AffiniPure Goat Anti-Rat IgG (1 : 15; Jackson ImmunoResearch Laboratories Inc.) for 5 hours in TBS with 1.5% milk and 0.25% Tween 20 at 4°C.

Immunolabelled sections were contrasted with uranyl acetate and Reynold's lead citrate and viewed on a transmission electron microscope (JEOL JEM-1011) operating at 60 kV. Electron micrographs were taken with a CCD camera (Morada; Olympus) and iTEM software (Olympus). A total of 15 randomly taken 25,000x magnification images of sections from four control and four ATS patient cell culture samples were evaluated by manually encircling relevant structures in ImageJ (NIH) and calculating their percentage of area relative to total cytoplasm. The distributions of gold particles over relevant structures were also counted. *P* values were calculated with Mann–Whitney's *U* test for pairwise comparison of nonnormal distribution data and nonparametric Kruskal-Wallis tests for multiple comparisons of nonnormal distribution data [21].

### 2.4. DNA Samples

Genomic DNA was isolated using the Blood & Cell Culture DNA Mini Kit (cat. No. 13323, Qiagen, Hilden, Germany) according to the manufacturer's instructions. The dsDNA samples were initially quantified using a NanoDrop spectrophotometer (Thermo Fisher Scientific Inc., Waltham, MA, USA) and normalized to 40 ng/*μ*l in TE buffer. Fragmentation was carried out in 0.25 ml of 40 ng/*μ*l stock DNA placed in 1.5 ml Eppendorf tubes immersed in an ice-water bath and sonicated for 6 × 30 sec by a Model 300 Sonic Dismembrator (Artek Systems Corp., Farmingdale, NY, USA). The evaluation of the size range of the fragmented DNA was performed by electrophoresis using a 1.5% agarose gel in TAE buffer (0.1 M Tris–HCl pH 8, 0.083 M acetic acid, 1 mM EDTA, and 0.5 mg/ml ethidium bromide).

### 2.5. Measurements of Global 5-Methylcytosine and 5-Hydroxymethylcytosine Levels in the Genome

Global levels of 5-mC and 5-hmC were assessed by MethylFlash Colorimetric DNA Quantification Kits from EpiGentek (cat. No. P-1030-48 and P-1032-48, Farmingdale, NY, USA). In these assays, genomic DNA was bound to specifically treated wells to have a high DNA affinity. The (hydroxy)methylated DNA fragments were detected with capture and detection antibodies following the company's protocol and quantified by reading the absorbance in a microplate spectrophotometer (Multiskan Spektrum, Thermo Fisher Scientific Inc., Waltham, MA, USA) at 450 nm. Liquid chromatography-tandem mass spectrometry (LC-MS/MS) was carried out using a previously published protocol [22] after hydrolyzing the genomic DNA to nucleobases with formic acid [23]. Briefly, 100 *μ*l of formic acid (100%) was added to 20 *μ*l of 40 ng/*μ*l DNA sample and pipetted into a 2 ml glass vial. The tightly crimped vial was kept at 130°C for 90 min. After nitrogen evaporation, the samples were reconstituted in acetonitrile solution containing 0.1% formic acid. Chemical standards dCTP, 5mdCTP, and 5hmdCTP were used to optimize the mass spectrometer (Sciex 6500 Qtrap System, Sciex, Redwood City, CA, USA). Separation was performed with a PerkinElmer Series 200 system (PerkinElmer, Waltham, MA, USA).

### 2.6. Selection of Candidate Gene Regions for Hydroxymethylation Status Analysis

Quantitative 5-mC- and 5-hmC-enrichment analyses were carried out on candidate genes coding for proteins involved in the metabolism or function of AA or described to be related to ATS. The selected genes were P3H1 (prolyl 3-hydroxylase 1), P4HA1 (prolyl 4-hydroxylase, alpha 1 subunit), P4HA2 (prolyl 4-hydroxylase alpha 2 subunit), PLOD1 (lysyl hydroxylase), ALDH1A1 (aldehyde dehydrogenase 1 family, member A1), and PPAR-*γ* (peroxisome proliferator-activated receptor gamma). Within these genes, specific regions were chosen for analyses based on highly modified cytosine levels in K562, HUVEC, HepG2, H1hESC, and Hela-S3 cell lines according to epigenome-wide data (e.g., Infinium HumanMethylation 450K microarray or reduced representation bisulfite sequencing), and H3K27 acetylation signal using ENCODE data shown in the UCSC Genome Browser [24]. Since 5-hmC is most abundant at both poised and active enhancers [25], we selected potentially active enhancer regions using H3K4 methylation and H3K27 acetylation marks [26]. In addition to the abovementioned genes, the GAPDH promoter was used as a negative control to check the lack of enrichment in the bound samples. PFKFB3 (6-phosphofructo-2-kinase/fructose-2,6-bisphosphatase 3) was included as a positive control based on the 5-hmC-sequencing signal of 3 publicly available datasets derived from cells of mesoderm origin (GSE64393, GSE49291, and GSE59989).

### 2.7. Measurements of Locus-Specific DNA Modification Level Changes after Ascorbate Treatment

Methylated DNA immunoprecipitation (MeDIP) was performed with Anti-5-Methylcytidine antibody (cat. No. BI-MECY-0500, Eurogentec, Liège, Belgium) using a previously established protocol [27] with a few technical changes. Shortly, 2 *μ*g (50 *μ*l of 40 ng/*μ*l conc.) of sonicated genomic DNA (input) was diluted to 100 *μ*l. A preclear step was performed with 30 *μ*l of Protein A/G (cat. No. 88803, Thermo Fisher Scientific Inc., Waltham, MA, USA) for 1 hour at 4°C on a rotator. Immunoprecipitation was carried out with 4 *μ*l of anti-5mC antibody. After the DNA-antibody incubation, 50 *μ*l of protein A/G was added to the tubes and incubated with the DNA-antibody mixture for 2 hours at 4°C. The beads were washed three times with 0.5 mL of IP buffer (0.14 M NaCl, 0.05% Triton X-100 in 10 mM PBS, pH 7), and then treated with proteinase K at 55°C overnight to elute the immunoprecipitated DNA (bound fraction), which was further purified with phenol-chloroform. Amplification of the input and MeDIP bound fractions was carried out with a GenomePlex Complete Whole Genome Amplification Kit (cat. No. WGA-2, Sigma-Aldrich, St. Louis, MO, USA) in order to carry out multiple reactions of gene-specific analyses.

Hydroxymethylated DNA immunoprecipitation (hMeDIP) was carried out with the Hydroxymethyl Collector Kit (cat. No. 55013, Active Motif, La Hulpe, Belgium) using 1.5 *μ*g of genomic DNA according to the manufacturer's instructions. Since the DNA yield in the hMeDIP bound samples was in the picogram range, the DNA concentration was determined with the QuantiFluor dsDNA System (Promega, Madison, WI, USA) on the Quantus Fluorometer (Promega, Madison, WI, USA). For the amplification, 30 pg of the hMeDIP bound fractions was used with the PicoPLEX WGA Kit (cat. No. E2620L, New England BioLabs Inc., Hitchin, Hertfordshire, UK). The WGA samples were purified with a GeneJET PCR Purification Kit (cat. No. K0702, Thermo Fisher Scientific Inc., Waltham, MA USA), measured with a NanoDrop spectrophotometer and diluted to 10 ng/*μ*l final concentration for qPCR.

Quantitative analyses of 5-mC and 5-hmC enrichment were carried out at specific regions of candidate genes. For region-specific primer pairs, see Table S1. The gene region-specific real-time PCR was performed on the amplified input and bound fractions using 20 ng of DNA per reaction and the PowerUp SYBR Green Master Mix (cat. No. A25742, Thermo Fisher Scientific Inc., Waltham, MA, USA) in 15 *μ*l volume on a QuantStudio 12K Flex Real-Time PCR System (Thermo Fisher Scientific Inc.). To determine the relative DNA enrichment, the comparative 2^−ΔΔCt^ method was used with postmortem dermis tissue as the reference sample (the first ΔCt was calculated from the difference of threshold cycle values of the Bound − Input samples).

### 2.8. Quantification of 5-Methylcytosine and 5-Hydroxymethylcytosine Level at a Specific CCGG Site

Locus-specific analysis was performed with the EpiMark 5-hmC and 5-mC Analysis Kit (cat. No. E3317S, New England BioLabs Inc., Hitchin, Hertfordshire, UK) according to the manufacturer's instructions. Briefly, DNA samples (5 *μ*g) were mixed with 31 *μ*l of 1x NEBuffer4 and 12.4 *μ*l of UDP-Glucose (final concentration 80 *μ*M). This mixture was split into two, and 30 units (3 *μ*l) of T4 *β*-glycosyltransferase was added to one of the tubes (the other tube served as a control reaction); both tubes were incubated at 37°C for 18 hours. After incubation, each reaction mixture was aliquoted into three tubes, labelled 1-3 of the T4 *β*-glycosyltransferase treatment, and 4-6 of the control reaction. The following restriction enzymes were added to the tubes: 100 U of *MspI* (can cleave if the internal C is 5-mC or 5-hmC but cannot cleave the glycosylated 5-hmC) was added to tubes 1 and 4 and 50 U of *HpaII* (can cleave only nonmodified C sequence) was added to tubes 2 and 5. No restriction enzymes were added to tubes 3 and 6 since they served as controls. Samples were incubated at 37°C for 8 hours. Afterwards, 20 *μ*g of proteinase K was added to each tube (final volume 53 *μ*l) and incubated at 40°C for 30 minutes. Finally, proteinase K was inactivated by incubating at 95°C for 10 minutes. The gene region-specific real-time PCR was performed using 2 *μ*l of each reaction mixture (~30 ng of DNA from tubes 1 to 6) in duplicates using the same PPAR-*γ* primers as described previously. The relative proportions of methylated and hydroxymethylated gene regions were calculated according to the formula provided by the manufacturer.

## 3. Results

### 3.1. Nuclear Accumulation of Ascorbate Diminished in ATS Fibroblasts

**Figure 1:**
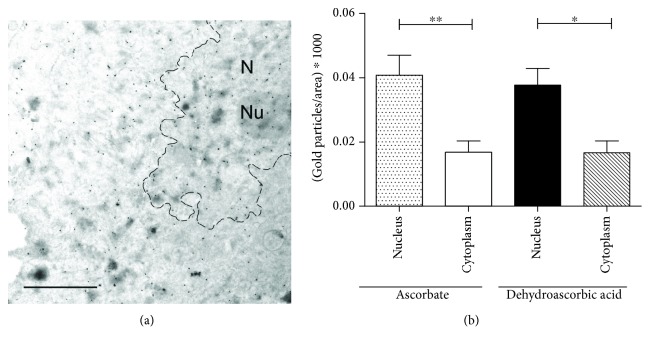
Subcellular distribution of ascorbate in human fibroblasts. Subcellular distribution of ascorbate in human fibroblasts derived from healthy controls (*n* = 4) was assessed by immunoelectron microscopy. After pretreatment of ascorbate or dehydroascorbic acid, cells were fixed and labeled with an antibody raised against ascorbate and analyzed following immunogold staining. (a) A representative image of the electron microscopy is shown. Scale bar is 1 *μ*m. N: nucleus, Nu: nucleolus. (b) Distribution of gold particles—representing ascorbate—was determined with respect to the relative area of the cytosol or the nucleus. A significantly higher level of the vitamin was observed in the nucleus compared to cytosol after both ascorbate and dehydroascorbate treatment. Data are means ± SEM, ^∗∗∗^ *p* < 0.001, ^∗∗^ *p* < 0.01.

While compartmentalization of AA is well established in plants, the compartment-specific levels of the vitamin are still unclear in mammalian cells. A previously developed method in plants based on high-resolution immunoelectron microscopy [28] was applied here to directly evaluate and simultaneously quantify subcellular AA levels. First, the subcellular distribution of AA was investigated in human fibroblasts isolated from healthy controls. As the basal AA level of human cells is far behind the AA content of plant cells, AA pretreatment of fibroblasts was applied to reach the detection level of the vitamin. In order to compare a possible difference in the uptake of the reduced and oxidized form of the vitamin, we applied both AA and DHA pretreatments. In both cases, similar and significant cellular accumulation of the vitamin was observed, eminently in the nucleus (Figure 1(a)). Nuclear AA level was significantly higher than that of the cytosol (Figure 1(b)), which implies the transport and consequent accumulation of the vitamin in the compartment. Gold particles indicating AA level in ER, mitochondria, and other smaller compartments were less abundant and hardly quantifiable separately.

**Figure 2:**
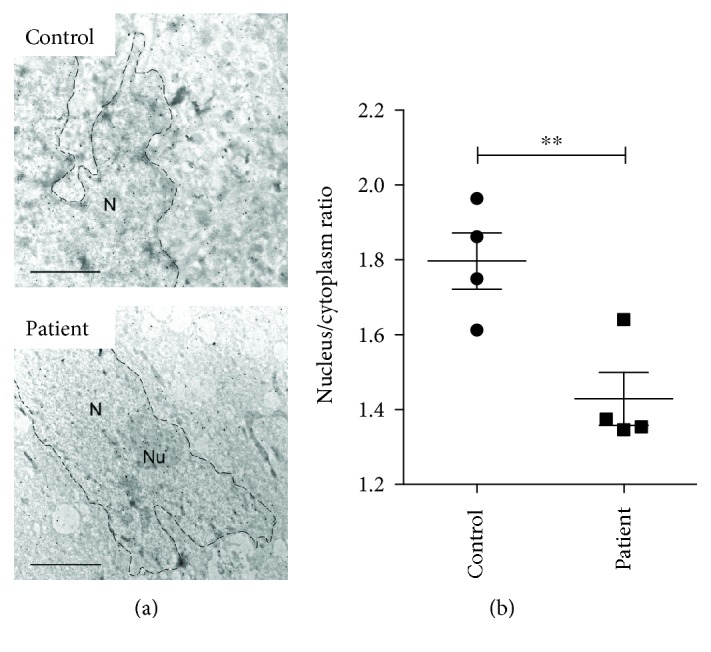
Diminished nuclear accumulation of vitamin C in ATS fibroblasts. Subcellular distribution of ascorbate of human fibroblasts derived from healthy controls or arterial tortuosity syndrome patients were compared by immunoelectron microscopy. After pretreatment of dehydroascorbic acid, cells were fixed and labeled with ascorbate antibody, and analyzed by following immunogold staining. (a) Representative images of the electron microscopy are shown from control (upper panel) and arterial tortuosity syndrome patient (lower panel) samples. Scale bar is 1 *μ*m. N: nucleus, Nu: nucleolus. (b) Nuclear/cytoplasmic ratio of immunogold particles—representing ascorbate—was assessed. A significantly lower level of nucleus/cytoplasm ascorbate ratio was found after dehydroascorbate treatment in fibroblasts derived from arterial tortuosity syndrome patients compared to control fibroblasts (4 in each groups). Data are means ± SEM, ^∗∗^ *p* < 0.01.

In a second set of experiments, AA levels of cytoplasm and nucleus were compared in control and ATS fibroblasts in a similar experimental setup (Figure 2(a)). We found a significantly lower nucleoplasmic/cytoplasmic ratio of AA in fibroblasts derived from ATS patients, indicating that defective transport to the nucleus indeed diminished nuclear AA levels (Figure 2(b)).

### 3.2. Global 5-Methylcytosine and 5-Hydroxymethylcytosine Levels in the Genome of Cultured Fibroblasts

**Figure 3:**
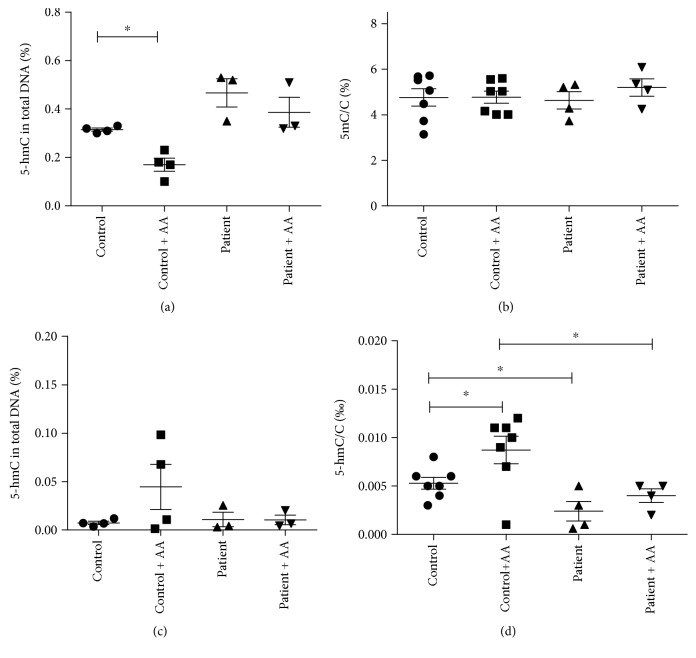
Global 5-methylcytosine and 5-hydroxymethylcytosine levels in the genome of fibroblasts derived from healthy controls and arterial tortuosity syndrome patients; 5-methylcytosine (5-mC) levels of fibroblasts derived from controls and arterial tortuosity syndrome patients, with or without ascorbate (AA) supplementation, measured with MethylFlash ELISA Colorimetric DNA kit (a) or with liquid chromatography-tandem mass spectrometry (LC-MS/MS, (b)). 5-Hydroxymethylcytosine (5-hmC) levels of control and patient fibroblasts, with or without AA supplementation, measured with ELISA kit (c) or LC-MS/MS (d). Data are presented as means ± SEM, *n* = 3-7, ^∗^ *p* < 0.05.

To examine whether GLUT10-deficient ATS fibroblasts possess any alteration at the epigenetic level due to defective nuclear DHA transport, we compared global levels of cytosine methylation and hydroxymethylation in cultured fibroblasts derived from controls and ATS patients. 5-mC and 5-hmC levels were compared before and after the addition of AA, which is expected to stimulate TET enzyme-mediated 5-mC-5-hmC conversion only in control cells. First, we used specific ELISA kits (Figures 3(a) and 3(c)), then we used liquid chromatography-tandem mass spectrometry (LC-MS/MS, Figures 3(b) and 3(d)). Analysis of the global DNA methylation level revealed no significant differences between ATS patients and controls (Figures 3(a) and 3(b)). In the control samples, AA treatment decreased the 5-mC level when measured by ELISA (*p* = 0.020); however, this result was not confirmed in the LC-MS/MS measurement. In the case of global DNA hydroxymethylation levels, ELISA measurement yielded quite low levels of 5-hmC (Figure 3(c)), showing that the kit was possibly not sensitive at this range of 5-hmC. The LC-MS/MS measurement yielded a more detailed picture (Figure 3(d)): hydroxymethylation levels in the genome of ATS fibroblasts were lower than that of controls (*p* = 0.027). Global 5-hmC levels (5-hmC/C ratios) were increased after AA treatment in both groups, but this increase was statistically significant only in the control group (*p* = 0.047).

### 3.3. Gene Region-Specific Cytosine Modifications

**Figure 4:**
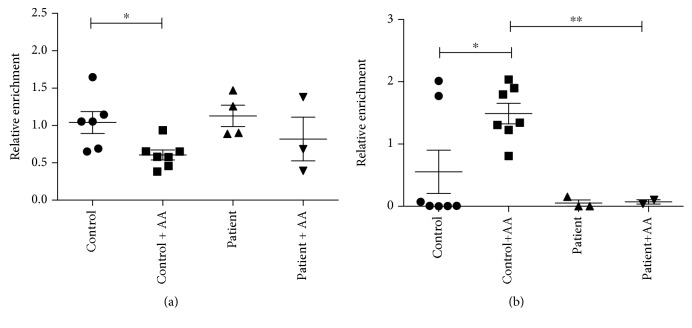
Gene region-specific cytosine modifications in peroxisome proliferator-activated receptor gamma gene using DNA immunoprecipitation enrichment. DNA immunoprecipitation was combined with quantitative PCR amplification (Q-MeDIP: (a), Q-hMeDIP: (b)) to analyse region-specific cytosine modifications in the selected gene region. The relative enrichment of the bound fractions of fibroblast DNA samples was compared to a control dermis tissue DNA sample. Data are means ± SEM, *n* = 3-7, ^∗^ *p* < 0.05.

DNA immunoprecipitation specifically designed to enrich 5-mC- and 5-hmC-containing regions were combined with quantitative PCR amplification of the bound and input fractions to analyse region-specific cytosine modifications. The relative enrichment of the bound fractions of the fibroblast samples was compared to the control (postmortem) dermis tissue's values. At the majority of the examined gene regions, decreased 5-hmC levels were observed in cultured fibroblasts derived from either controls or patients as compared to the tissue level, which is in line with previous observations [29], but this decrease could be reversed by ascorbate treatment (Table S1). Due to the limited number of patients and the high variability of the qPCR values, these changes were significant only in one gene. In the case of PPAR-*γ*, we observed a significantly higher 5-hmC level in the control group after AA treatment compared to untreated control samples (Figure 4(b)). AA treatment of fibroblasts derived from ATS patients had no effect on the 5-hmC level. A similar reversal of the 5-hmC level after AA treatment was observed at the 5-hmC-control region in the *PFKFB3* gene (Table S1). Furthermore, AA treatment significantly decreased the 5-mC level of the selected region in PPAR-*γ* only in control fibroblasts, potentially indicating increased active demethylation processes (Figure 4(a)).

**Figure 5:**
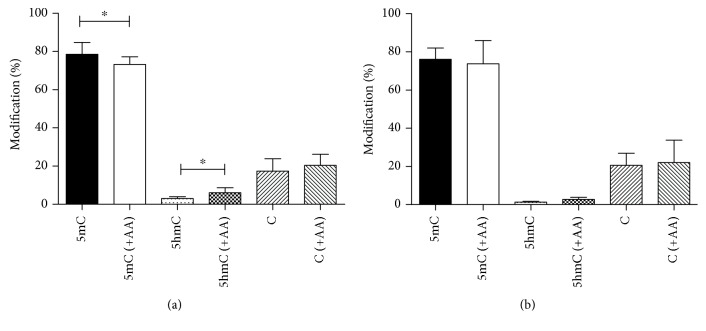
Gene region-specific cytosine modifications in peroxisome proliferator-activated receptor gamma gene using differential restriction endonuclease cleavage. The EpiMark 5-hmC and 5-mC Analysis Kit uses a glycosylation pretreatment to distinguish 5-hmC from 5-mC via differential restriction endonuclease digestion of a CCGG sequence. The relative ratios (percentage of 5-mC, 5-hmC, and non-modified C) are quantified using quantitative PCR amplification. Average values ± SEM of modified C % are shown for controls ((a), *n* = 6) and patients ((b), *n* = 3), ^∗^ *p* < 0.05.

To reinforce this result, we carried out another locus-specific measurement on the same PPAR-*γ* gene region using the EpiMark 5-hmC and 5-mC Analysis Kit. This restriction enzyme-based technique distinguishes 5-mC from 5-hmC by the glycosylation of the hydroxyl group of 5-hmC. When the internal C of a CCGG sequence is modified, the glycosylation of 5-hmC changes the *MspI* cleavage site into a noncleavable one (for details, see Materials and Methods). The AA treatment decreased 5-mC levels (*p* = 0.029) and increased 5-hmC levels (*p* = 0.026) in control fibroblast samples, while methylation and hydroxymethylation status in ATS patient fibroblasts did not change significantly (Figure 5).

## 4. Discussion

The presented results demonstrate that the intracellular compartmentalization of vitamin C can be an important factor in epigenetic regulation. We used ATS fibroblasts as a model system: ATS is a genetic disease due to the mutations in *SLC2A10* gene, encoding GLUT10, a glucose/DHA transporter of the endomembranes. As we previously reported, intracellular DHA transport was impaired in these cells [17]. In the present study, we demonstrate that in ATS fibroblasts (i) DNA methylation cannot be decreased by AA addition (while in control fibroblasts, it can be); (ii) the hydroxymethylation level is lower than in control cells; (iii) unlike in control fibroblasts, the hydroxymethylation level cannot be significantly increased by AA addition; (iv) decreased hydroxymethylation of a specific gene region in PPAR-*γ* gene is present; and (v) AA addition cannot stimulate 5-mC-to-5-hmC conversion in the PPAR-*γ* gene unlike in control fibroblasts.

In our first exploratory experiment, the nucleoplasmic occurrence of vitamin C was checked. Hitherto, the presence of vitamin C in the nucleus has not been directly measured in living animal cells. By using immunogold labelling and transmission electron microscopy, a technique successfully applied in plant cells [28], we verified that vitamin C appeared and accumulated in the nucleus of control fibroblasts upon AA or DHA addition within 5 minutes. When nuclear vitamin C uptake was compared in control and ATS fibroblasts, we observed a significantly lower ratio of nucleoplasmic versus cytoplasmic vitamin C concentrations upon DHA addition. Taking into account that vitamin C uptake and its total intracellular concentration is lower in ATS fibroblasts, one can assume a serious nucleoplasmic impoverishment in vitamin C in ATS fibroblasts.

Secondly, global methylation and hydroxymethylation of genomic DNA was analysed by ELISA. One of the most thoroughly investigated epigenetic marks is the 5-methylation of the cytosine base in dsDNA. In mammalian somatic cells, 5-mC occurs mainly in CG dinucleotides. The regulatory effects of 5-mC translating the environmental clues are well established [30]. The oxidative modification of 5-mC received new interest in the last decade. Growing evidence shows the potential role of the 5-hmC signal in gene expression, which is present mostly in gene bodies and promoter regions in human tissues, e.g., in the brain [31], chondrocytes [32], breast, and liver [33]. The conversion of 5-mC to 5-hmC—catalyzed by TET enzymes—is AA sensitive, as it has been shown in cultured mouse embryonic stem cells [34] and fibroblasts [29]. The 5-mC level measured in ATS fibroblasts could not be modified by AA administration; however, the 5-mC level in control cells could be diminished by AA treatment. The detection of global 5-hmC levels did not result in unequivocal data, probably due to the low sensitivity of the ELISA method. Therefore, the 5-hmC levels were estimated by a LC-MS/MS technique, which showed that the hydroxymethylation level in the genome of patients were lower than those in controls. Global 5-hmC levels (5-hmC/C ratios) were increased after AA treatment in both groups, but this increase was statistically significant only in the control cells.

In the next step, quantitative 5-mC- and 5-hmC-enrichment analyses were carried out on candidate genes, which code for proteins required in AA metabolism or function or genes related to ATS pathogenesis. PPAR-*γ* has a key role in the regulation of extracellular matrix formation. We found lower hydroxymethylation of the PPAR-*γ* gene in fibroblasts from ATS patients. While in control cells 5-hmC formation could be stimulated by AA addition, this treatment was ineffective in patients' cells. The decreased methylation level upon AA addition in control cells confirmed the observation. These results can be ascribed to a decreased accessibility of the cofactor to DNA demethylases.

The involvement of the epigenetic regulation of the PPAR-*γ* gene is not unprecedented. TET upregulation led to reduced DNA methylation and elevated 5-hmC formation, both globally and specifically at the PPAR-*γ* locus in preadipocytes, which was required for adipogenic differentiation [35]. Epigenetic regulation of the antifibrogenic PPAR-*γ* and the profibrogenic TGF-*β*1 was observed during wound healing [36]. Moreover, hypermethylation of the promoter of PPAR-*γ* gene was significantly associated with fibrosis in the liver [37]. Activation of PPAR-*γ* has been reported to inhibit the TGF-*β* pathway in mesangial cells and mesenchymal stem cells [38, 39]. These observations can be related to the pathomechanism of ATS. Increased TGF-*β* activity has also been repeatedly postulated as an important causal factor of genetic arteriopathies [40]. Dysregulation of the PPAR-*γ*-TGF-*β* pathway has been reported in fibroblasts from ATS patients [41]. Indeed, PPAR-*γ* activators are among the candidates for the treatment of aorta aneurysms [42].

**Figure 6:**
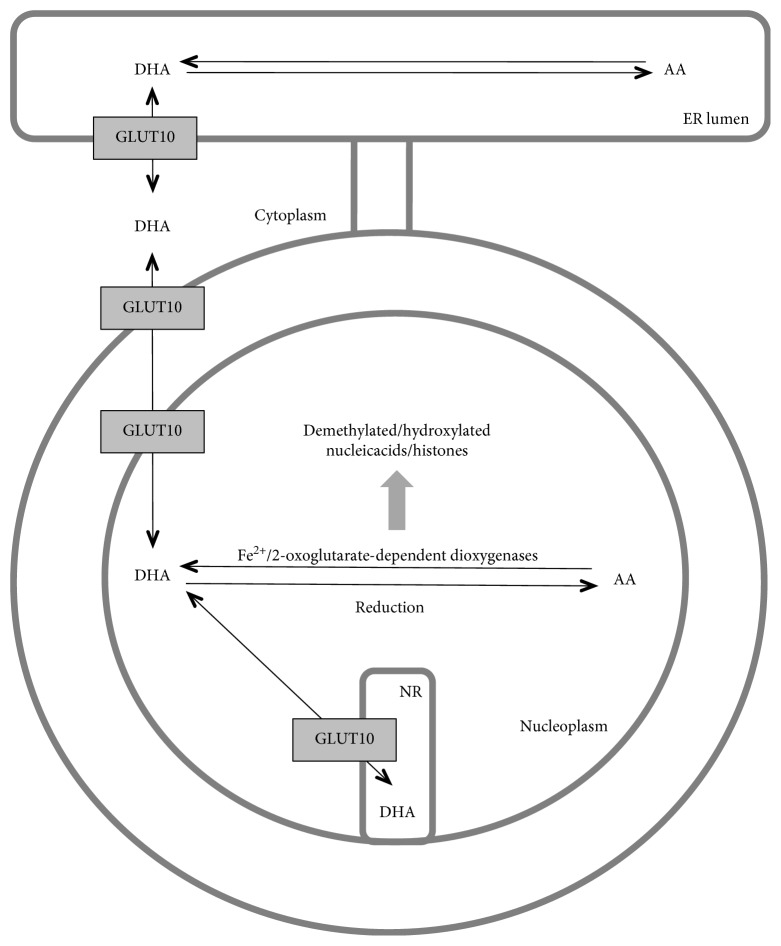
Compartmentalization-dependent effect of vitamin C on epigenetic regulation. Vitamin C is transported as dehydroascorbic acid (DHA) by GLUT10 into the endoplasmic reticulum (ER) lumen and in the nucleoplasm. Having been reduced to ascorbate (AA), it can serve as a cofactor for Fe^2+^/2-oxoglutarate-dependent dioxygenases, catalyzing (among others) histone and DNA demethylation. NR, nucleoplasmic reticulum.

The role of vitamin C in epigenetic regulation has been demonstrated by several studies in different experimental systems (for recent reviews, see [5, 43]). Ascorbate concentration seems to be a rate-limiting factor in the catalytic activity of the histone and DNA demethylases of the Fe^2+^/2-oxoglutarate-dependent dioxygenase family [44]. Plasma membrane vitamin C transporters have emerged as modulators of ascorbate-dependent epigenetic regulation [45–47]. The present paper demonstrates firstly that intracellular vitamin C transporters responsible for the entry of AA or DHA into subcellular compartments are also important determinants of vitamin C effects. The absence of GLUT10, a DHA transporter of the endomembranes, hinders vitamin C transport into the nucleoplasm and the lumen of the ER membrane (Figure 6). A lower AA concentration in the nucleus results in the suboptimal functioning of Fe^2+^/2-oxoglutarate-dependent dioxygenases and increased 5-mC/5-hmC ratio.

Subcellular or noncanonical scurvy has already been reported in the ER lumen [48, 49], which was due to the increased consumption of AA. Here, we showed that the decreased entry could also cause AA deficiency in another compartment, i.e., the nucleus, resulting in epigenetic alterations. Further studies are needed to clarify whether these changes significantly contribute to the pathogenesis of ATS or not. In any case, increasing global DNA methylation has been reported in any tissue across the entire life course [50]. The accelerated epigenetic clock in ATS fibroblasts may lead to premature aging with its undesirable consequences. Furthermore, gene-specific alterations in the methylation (e.g., of PPAR-*γ* gene) can also support a more specific hypothesis for the pathogenesis.

## 5. Conclusions

Emerging evidence suggests that vitamin C can influence gene expression via regulating epigenetic processes. Here, we report that decreased nuclear accumulation of ascorbate in GLUT10-deficient fibroblasts of ATS patients is accompanied with an altered DNA hydroxymethylation pattern both at the global level and at specific gene regions. The findings suggest that vitamin C has an epigenetic role in the pathomechanism of ATS and represents the first example for the role of intracellular vitamin C transport in epigenetic regulation. The results suggest that ATS can be regarded as a compartmentalization disease.

## Data Availability

All of the data used to support the findings of this study are included within the article.

## Abbreviations

5-hmC: 5-hydroxymethylcytosine
5-mC: 5-methylcytosine
AA: Ascorbate
ALDH1A1: Aldehyde dehydrogenase 1 family, member A1
ATS: Arterial tortuosity syndrome
DHA: Dehydroascorbic acid
ER: Endoplasmic reticulum
GAPDH: Glyceraldehyde 3-phosphate dehydrogenase
hMeDIP: Hydroxymethylated DNA immunoprecipitation
LC-MS: Liquid chromatography-tandem mass spectrometry
MeDIP: Methylated DNA immunoprecipitation
P3H1: Prolyl 3-hydroxylase 1
P4HA1: Prolyl 4-hydroxylase, alpha 1 subunit
P4HA2: Prolyl 4-hydroxylase alpha 2 subunit
PFKFB3: 6-phosphofructo-2-kinase/fructose-2,6-biphosphatase 3
PLOD1: Lysyl hydroxylase
PPARG: Peroxisome proliferator-activated receptor gamma
TET: Ten-eleven translocation.

## Supplementary Materials

Supplementary Materials Table S1: quantitative analyses of 5-mC and 5-hmC enrichment of specific regions of candidate genes.
